# 高效液相色谱法快速同时检测全血中两种免疫抑制剂

**DOI:** 10.3724/SP.J.1123.2022.03033

**Published:** 2023-02-08

**Authors:** Yongpeng HUANG, Hui TANG, Xiangyan MENG, Hui ZHONG, Yunyang SONG, Bo CHEN, Zhiyun ZOU

**Affiliations:** 国民核生化灾害防护国家重点实验室，北京 102205; State Key Laboratory of NBC Protection for Civilian，Beijing 102205，China

**Keywords:** 高效液相色谱, 免疫抑制剂, 环孢素A, 西罗莫司, 生物液相色谱柱, high performance liquid chromatography （HPLC）, immunosuppressant, cyclosporine A, sirolimus, biological liquid chromatography column

## Abstract

环孢素A和西罗莫司是许多器官移植手术中广泛使用的免疫抑制剂，且一起使用时会产生协同效应，但这两种免疫抑制剂的治疗窗口都非常窄，仅在特定的血药浓度范围内有预期的治疗效果。因此，快速同时检测全血中这两种免疫抑制剂的浓度，可为患者器官移植手术后的给药方案提供有价值的信息。该工作首先考察了环孢素A和西罗莫司在生物液相色谱柱和传统液相色谱柱上的色谱行为，然后基于生物液相色谱柱，建立了可快速分离和检测全血中环孢素A和西罗莫司的高效液相色谱分析方法。全血样品经样品前处理后进样分析，采用ZORBAX 300SB C8柱（250 mm×4.6 mm， 5.0 μm）进行分离，以乙腈-水（70∶30， v/v）为流动相进行等度洗脱，柱温为60 ℃，流速为1.0 mL/min，检测波长为205 nm和278 nm，进样量为20 μL。结果表明，环孢素A和西罗莫司在6 min内可实现较好的分离；环孢素A和西罗莫司在各自的浓度范围内具有良好的线性关系（*r*>0.997），检出限（*S/N*=3）分别为10 ng/mL和1 ng/mL，定量限（*S/N*=10）分别为30 ng/mL和2 ng/mL， 3个水平的平均加标回收率分别为83.5%~89.7%和95.8%~97.8%，相对标准偏差（RSD）分别为3.2%~9.0%和3.4%~6.7%（*n*=5）。该方法操作简便，流动相简单，分析时间短，线性范围宽，灵敏度高，可用于全血中环孢素A和西罗莫司的含量检测。

## Abstract (en)

Cyclosporine A and sirolimus are immunosuppressants that are widely used in many organ transplantation procedures. They exhibit some complementary mechanisms of action and interact synergistically when used together. However， they are critical-dose drugs and have a narrow therapeutic index. They provide the desired therapeutic effect with acceptable tolerability only within a specific range of blood concentrations. Therefore， the rapid and simultaneous detection of the concentrations of cyclosporine A and sirolimus in whole blood could provide valuable information on planning medicine administration after organ transplantations. In this study， firstly， the chromatographic behaviors of cyclosporine A and sirolimus on a biological liquid chromatography （BioLC） column and traditional liquid chromatography （TraLC） columns were investigated systematically under the same chromatographic conditions. The results suggested that the peak height and peak width of cyclosporine A and sirolimus on the BioLC column， ZORBAX 300SB C8 （250 mm×4.6 mm， 5.0 μm）， were the highest and narrowest， respectively. The number of theoretical plates of cyclosporine A and sirolimus on the ZORBAX 300SB C8 column increased significantly when the volume ratio of acetonitrile in the mobile phases was greater than 70%. Their retention time on the BioLC and TraLC columns was negligibly affected by the use of formic acid and trifluoroacetic acid as the mobile phases. In the range of the experimental column temperature， the number of theoretical plates of cyclosporine A and sirolimus on the ZORBAX 300SB C8 column was significantly higher than that on the two TraLC columns. Furthermore， the relationship between the retention factor and column temperature of cyclosporine A on the ZORBAX 300SB C8 column was different from that on the two TraLC columns. Subsequently， a high performance liquid chromatography method based on the ZORBAX 300SB C8 column was established for the rapid separation and determination of cyclosporin A and sirolimus in whole blood. A sample of whole blood with a volume of 50 μL was prepared by protein precipitation with 1 mol/L sodium hydroxide and then extracted into 500 μL of ether-methanol （95∶5， v/v）. After centrifugation at 14000 r/min for 10 min， the organic layer was removed and evaporated under a stream of nitrogen at 50 ℃. The residue was then reconstituted in 200 μL of methanol for use. Cyclosporin A and sirolimus were separated through isocratic elution on the ZORBAX 300SB C8 column. The column temperature was set at 60 ℃. The mobile phase was acetonitrile-water （70∶30， v/v） and the flow rate was 1.0 mL/min. The detection wavelengths were 205 nm for cyclosporine A and 278 nm for sirolimus. The injection volume was 20 μL. The external standard method was used to quantify cyclosporine A and sirolimus. Under the optimized conditions， cyclosporine A and sirolimus were well-separated within 6 min with a resolution of 3.7 at 205 nm. In addition， the endogenous substances in whole blood negligibly interfered in the detection of sirolimus， while two endogenous substances slightly affected the detection of cyclosporine A. Cyclosporine A and sirolimus both showed good linear relationships in their respective concentration （*r*>0.997）. The limits of detection （LODs） of cyclosporine A and sirolimus were respectively calculated to be 10 ng/mL and 1 ng/mL based on a signal-to-noise ratio of 3， and the limits of quantification （LOQs） were 30 ng/mL and 2 ng/mL based on a signal-to-noise ratio of 10. In the whole blood samples， the recoveries of cyclosporine A and sirolimus at three spiked levels were in the ranges of 83.5%-89.7% and 95.8%-97.8% with relative standard deviations （RSDs） of 3.2%-9.0% and 3.4%-6.7% （*n*=5）， respectively. The established method is simple in operation， can be performed with a simple mobile phase， has a short analysis time， and provides a wide linear range and high sensitivity； hence， it can be applied to the determination of cyclosporine A and sirolimus in whole blood.

环孢素A和西罗莫司（化学结构见图1）作为免疫抑制剂，已成功应用于许多器官移植手术^[1⇓-3]^。这两种免疫抑制剂的治疗窗口非常窄，仅在特定的血药浓度范围内有预期的治疗效果，当血药浓度较低时，存在器官排斥的风险，而血药浓度较高时，可能会出现肾脏毒性、心脏毒性、神经毒性等严重的副作用^[4]^。另一方面，这两种免疫抑制剂一起使用时会产生协同效应，临床上通常将其联合使用^[5]^。免疫抑制剂的治疗范围受移植器官、患者年龄等多种因素影响。据报道，环孢素A的治疗范围为50~300 ng/mL^[6]^，西罗莫司的治疗范围为12~30 ng/mL，当与环孢素A联合使用时，其治疗范围为4~12 ng/mL^[5]^。因此，对这两种免疫抑制剂同时进行血药浓度监测，在器官移植后的给药方案中发挥着重要作用。

**图1:**
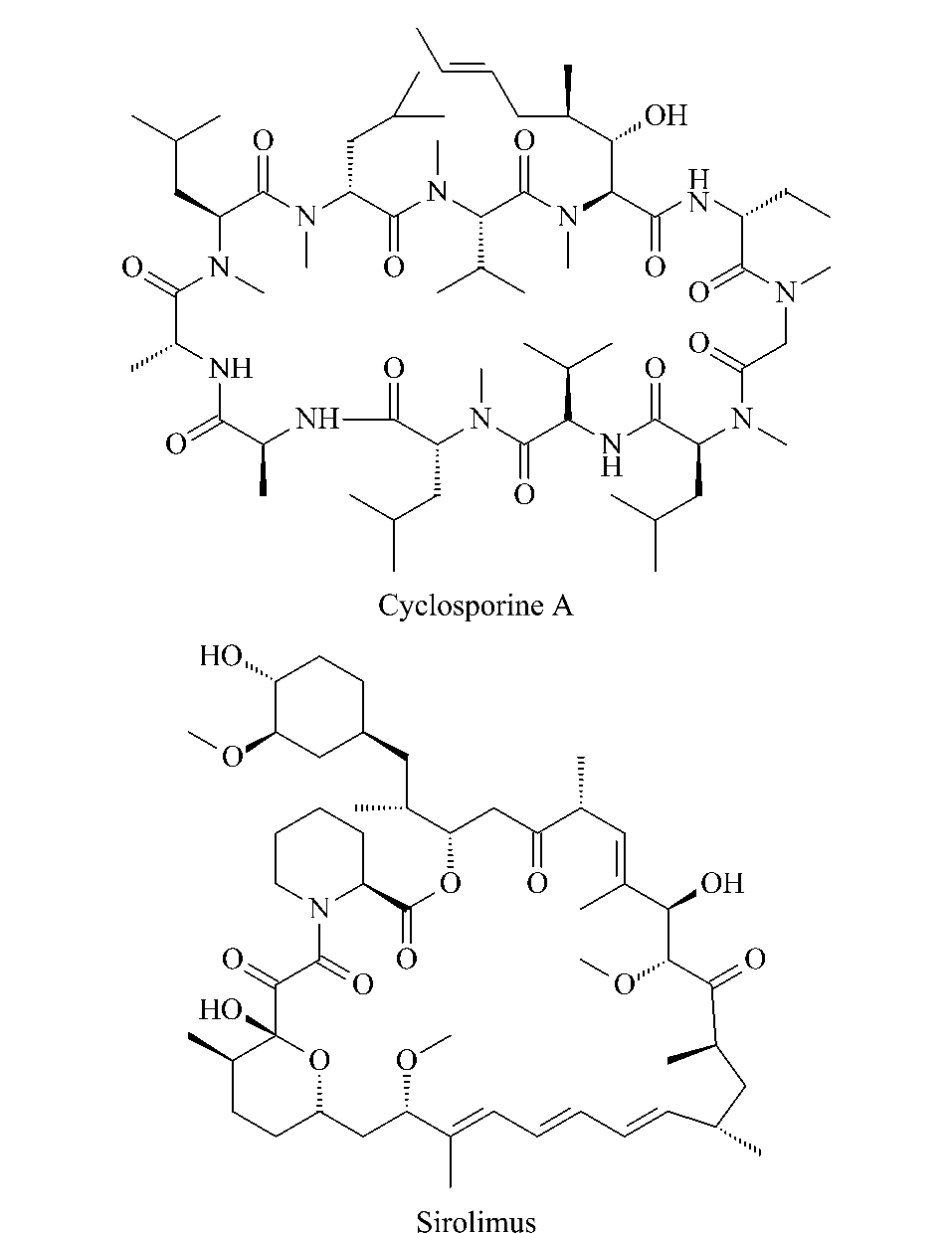
免疫抑制剂的化学结构式

文献报道的用于定量分析环孢素A、西罗莫司、他克莫司等免疫抑制剂的方法主要包括免疫分析法^[7]^、高效液相色谱法（HPLC）^[8]^和高效液相色谱-串联质谱法（HPLC-MS/MS）^[9⇓⇓-12]^。免疫分析法操作简单，但抗体可能会与免疫抑制剂代谢的非活性产物发生交叉反应，从而高估药物水平^[13]^。HPLC也常用于对全血中单个或多个免疫抑制剂的定量分析，通常需要较长的色谱运行时间，以分离多个目标物和减小基质干扰^[14]^；最近报道了一种基于智能化学计量学的高效液相色谱法，该法通过对紫外光谱进行处理，实现了快速定量分析全血中3种免疫抑制剂^[15]^。HPLC-MS/MS以独特的选择性和灵敏度，主要用于检测动物内脏、血液、尿液等生物样品中免疫抑制剂的含量，但该方法也受到基质效应、仪器成本高和需要专业人员操作等因素的限制^[16]^。

由于环孢素A的构象异构体在传统反相C8或C18柱上具有一定的分离度（*R*），造成其具有明显的色谱峰展宽效应^[17]^，为了达到更好的分离效果和更高的检测灵敏度，氰基柱^[18]^或苯基-己基柱^[10,19]^成为分离和检测这些免疫抑制剂更佳的选择；此外，采用HPLC快速分离和检测全血中环孢素A和西罗莫司含量的文献也少有报道。随着色谱柱技术的发展，针对蛋白质、多肽等大分子的生物液相色谱柱不断研发出来，本研究考察了环孢素A和西罗莫司在生物液相色谱柱和传统液相色谱柱上的色谱行为，并基于生物液相色谱柱建立了可快速分离和检测全血中环孢素A和西罗莫司的HPLC分析方法。

## 1 实验部分

### 1.1 仪器、试剂与材料

Agilent 1260 Infinity Ⅱ高效液相色谱仪，配有二极管阵列检测器（DAD）； XP105天平（上海Mettler-Toledo公司）。

乙腈和甲醇（HPLC级，德国Merck公司）；甲酸、三氟乙酸、乙醚和氢氧化钠（分析纯，上海Macklin Biochemical公司）；环孢素A和西罗莫司（纯度≥98%，南京都莱生物技术有限公司）；实验用水为超纯水。

### 1.2 标准溶液的配制

标准储备溶液：分别准确称取适量的环孢素A和西罗莫司，用甲醇溶解并定容，分别配制成1000 μg/mL和500 μg/mL的标准储备液，于4 ℃避光保存。

混合标准储备溶液：取各标准储备溶液适量，用甲醇稀释得混合标准储备溶液，现用现配。

基质标准工作溶液：用经样品前处理后的空白基质逐级稀释混合标准储备液，制备成基质标准工作溶液，现用现配。

### 1.3 样品前处理方法

准确量取50 μL全血样品，加入100 μL氢氧化钠水溶液（1 mol/L）并振荡30 s，再加入500 μL乙醚-甲醇（95∶5， v/v）并振荡30 s，然后以14000 r/min离心10 min，取有机层溶液在50 ℃下用氮气吹干，最后加入200 μL甲醇溶解，待用。

### 1.4 液相色谱条件

色谱柱：ZORBAX 300 SB C8色谱柱（250 mm×4.6 mm， 5.0 μm，美国Agilent公司）；柱温60 ℃；流动相：乙腈-水（70∶30， v/v）；流速1.0 mL/min；检测波长：205 nm（环孢素A）和278 nm（西罗莫司）；进样量20 μL。

## 2 结果与讨论

### 2.1 色谱条件对免疫抑制剂色谱行为的影响

实验中，保持环孢素A和西罗莫司溶液浓度一致，在流速为1 mL/min、进样量为20 μL和检测波长分别为205 nm和278 nm的条件下，分别考察了色谱柱、流动相组成、流动相添加剂和柱温对两种免疫抑制剂色谱行为的影响。

#### 2.1.1 色谱柱

在流动相为乙腈-水（80∶20， v/v）、柱温为50 ℃的条件下，考察了环孢素A和西罗莫司在6种Agilent反相色谱柱（色谱柱基本信息见表1）上的色谱保留行为，色谱图和半峰宽值见图2。结果表明，环孢素A和西罗莫司在不同色谱柱上的色谱保留行为差异显著，尤其环孢素A在不同色谱柱上的色谱峰展宽效应明显不同；与传统液相色谱柱相比，两种免疫抑制剂均在ZORBAX 300SB C8生物液相色谱柱上具有最高的色谱峰和最小的半峰宽，尤其环孢素A在该柱的色谱峰高和半峰宽分别是其他5种色谱柱的2.5~7.5倍和0.13~0.39倍。可以看出，采用生物液相色谱柱对环孢素A和西罗莫司进行分析，可显著提高检测灵敏度。这可能与色谱柱的孔径有关，ZORBAX 300SB C8柱的孔径最大，其次为InfinityLab Poroshell 120 EC C8柱，最小的为ZORBAX Eclipse XDB C18柱，两种免疫抑制剂的色谱峰高和半峰宽的变化趋势也基本与色谱柱的孔径变化分别呈正相关和负相关。

**表1:** 使用的色谱柱基本信息

No.	Column style	Model	Specification	Maximum operating temperature/ ℃	Status
1	traditional	ZORBAX Eclipse XDB C18	250 mm×4.6 mm，5.0 μm，8 nm	60	used
2	traditional	ZORBAX Eclipse Plus C18	250 mm×4.6 mm，5.0 μm，9.5 nm	60	used
3	traditional	ZORBAX Eclipse Plus C8	250 mm×4.6 mm，5.0 μm，9.5 nm	60	new
4	traditional	ZORBAX Eclipse PAH	250 mm×4.6 mm，5.0 μm，9.5 nm	60	new
5	traditional	InfinityLab Poroshell 120 EC C8	150 mm×4.6 mm，2.7 μm，12 nm	60	new
6	biological	ZORBAX 300SB C8	250 mm×4.6 mm，5.0 μm，30 nm	80	new

**图2:**
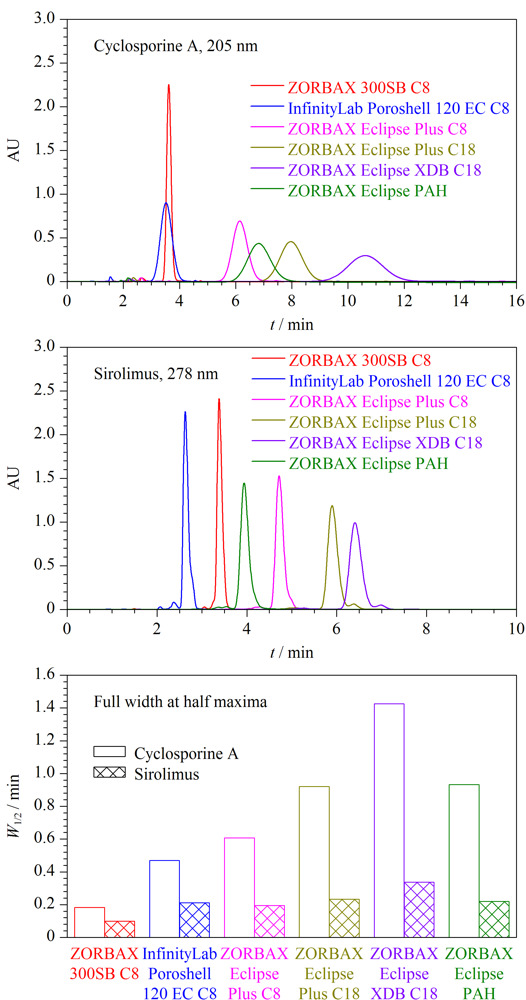
环孢素A和西罗莫司在不同色谱柱上的色谱图和半峰宽

#### 2.1.2 流动相组成

在50 ℃柱温下，考察了乙腈-水流动相体系对环孢素A和西罗莫司在3种色谱柱上色谱行为的影响，流动相组成与保留因子（*k*）和理论塔板数（*N*）的关系见图3。结果表明，在3种色谱柱上，环孢素A和西罗莫司的保留时间（*t*_R_）均随着流动相中乙腈体积分数的减小呈指数增大的趋势；当流动相中乙腈的体积分数大于70%时，环孢素A和西罗莫司在ZORBAX 300SB C8柱上的理论塔板数均随着乙腈体积分数的增大而显著增大，且显著高于在其他两种色谱柱上的理论塔板数。

**图3:**
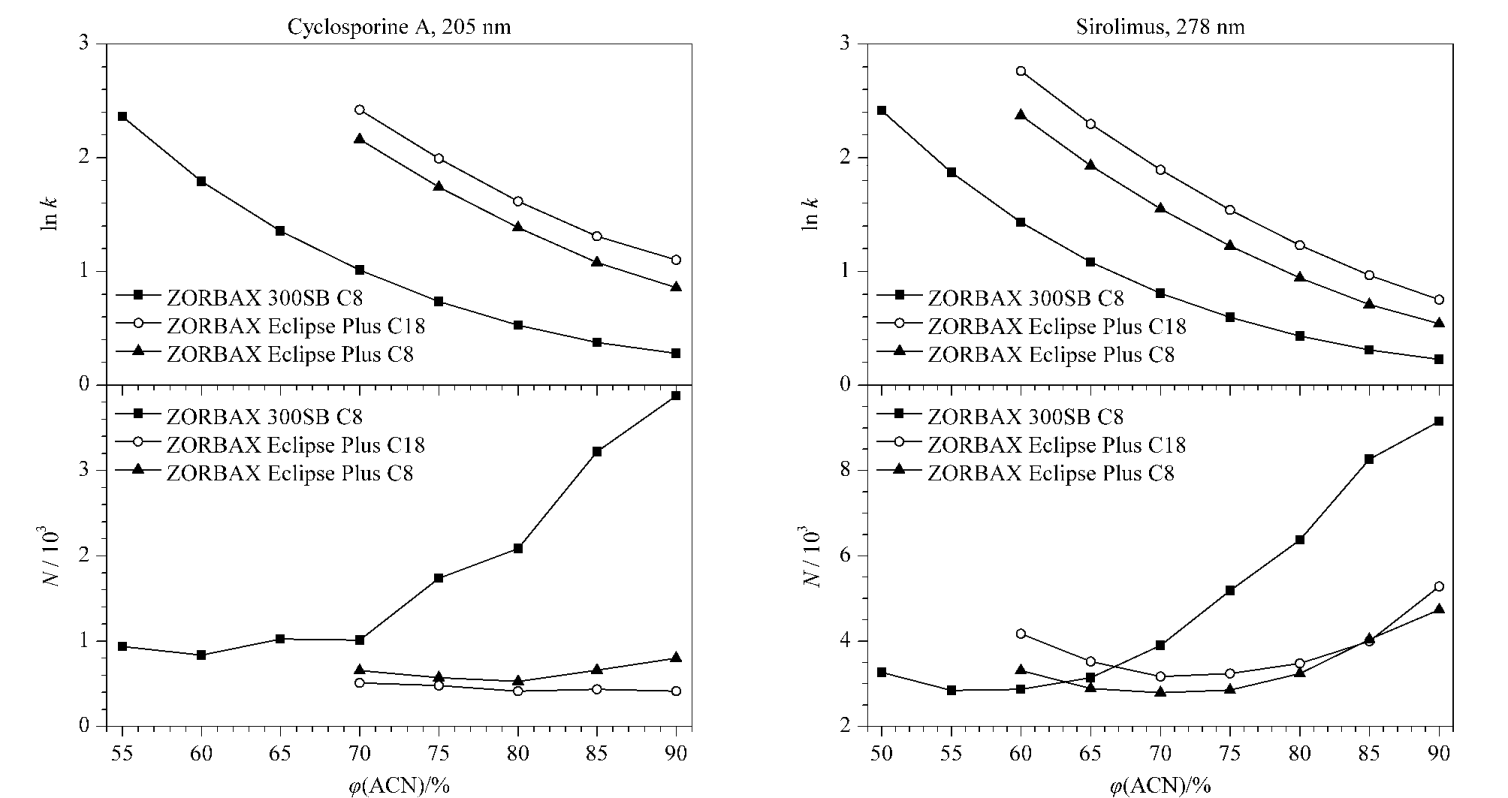
环孢素A和西罗莫司在乙腈-水流动相体系中的色谱峰参数

#### 2.1.3 流动相添加剂

在50 ℃柱温条件下，通过在流动相中添加三氟乙酸和甲酸两种离子对试剂，考察了其对环孢素A和西罗莫司保留时间的影响，结果见图4。结果表明，在3种色谱柱上，两种流动相添加剂对环孢素A和西罗莫司的保留时间几乎没有影响。这是由于环孢素A和西罗莫司化学结构中均没有游离的氨基和羧基，故酸性离子对试剂对其色谱保留行为没有显著影响。

**图4:**
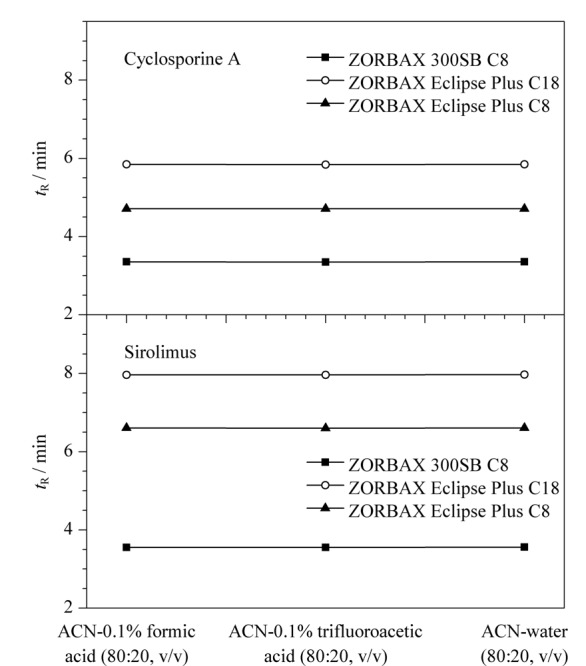
环孢素A和西罗莫司在不同流动相中的保留时间

#### 2.1.4 柱温

在流动相为乙腈-水（80∶20， v/v）的条件下，采用Van’t Hoff方程^[20⇓-22]^考察了柱温对环孢素A和西罗莫司色谱行为的影响，柱温与保留因子*k*和理论塔板数*N*的关系见图5和图6。结果表明，随着柱温的升高，在3种色谱柱上，环孢素A和西罗莫司的保留因子基本呈减小趋势；在实验柱温范围内，西罗莫司在3种色谱柱上的ln*k*和柱温的倒数（1/*T*）线性关系均较好（*r*>0.96），环孢素A在ZORBAX Eclipse Plus C18和ZORBAX Eclipse Plus C8柱上相应的线性关系较好（*r*>0.99）；当柱温在45~70 ℃ （1/*T*： 3.14×10^-3^~ 2.91×10^-3^ K^-1^）之间时，环孢素A在ZORBAX 300SB C8柱上相应的线性关系较好（*r*>0.99），而柱温在25~40 ℃ （1/*T*： 3.35×10^-3^~ 3.19 ×10^-3^ K^-1^）之间时，其线性关系较差，且柱温为25 ℃（1/*T*： 3.35×10^-3^ K^-1^）时色谱峰发生裂分现象；在实验柱温范围内，环孢素A和西罗莫司在ZORBAX Eclipse Plus C18和ZORBAX Eclipse Plus C8柱的理论塔板数相近，而在其ZORBAX 300SB C8柱的理论塔板数均显著高于其他两柱。有理论^[17]^认为，升高柱温可以减小免疫抑制剂的构象分离和色谱峰宽，从而提高柱效，并且升高柱温可减小流动相的黏度，有利于减小流动相传质阻力，可进一步提高柱效；此外，ZORBAX 300SB C8柱具有更大的孔径，固定相传质阻力也更小，柱效也更高。因此，在较高的柱温条件下，环孢素A和西罗莫司在ZORBAX 300SB C8柱上的柱效和检测灵敏度会显著优于其他两柱。

**图5:**
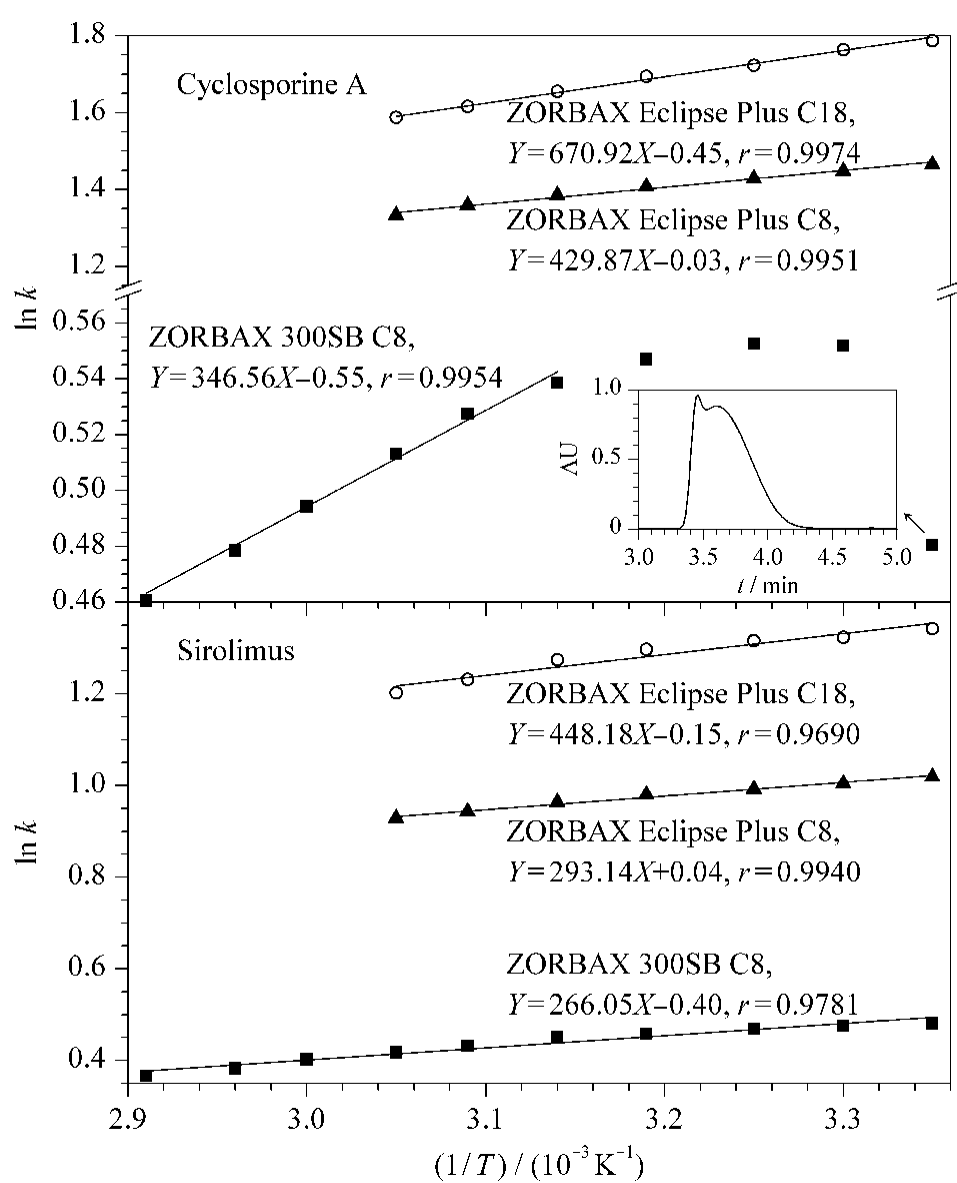
环孢素A和西罗莫司在不同色谱柱上的保留因子与柱温的关系图

**图6:**
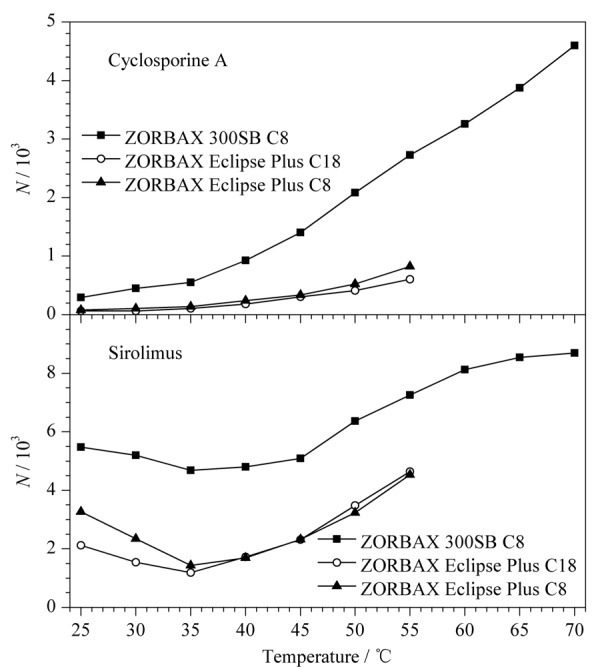
环孢素A和西罗莫司在不同色谱柱上的柱温与柱效关系图

### 2.2 液相色谱条件优化

生物液相色谱柱和传统液相色谱柱对两种免疫抑制剂色谱行为的影响结果表明，生物液相色谱柱在检测环孢素A和西罗莫司时具有显著的优势，因此，在ZORBAX 300SB C8柱上对两种免疫抑制剂分离的色谱条件进行优化，不同色谱条件下的分离度结果见图7。结果表明，分离度随流动相中乙腈体积分数的减小而增大，当乙腈体积分数小于80%时，环孢素A和西罗莫司的色谱峰可完全分离（*R*≥ 1.5）；分离度随柱温的升高而增大，当柱温高于50 ℃时，环孢素A和西罗莫司的色谱峰可完全分离。因此，减小流动相中乙腈的体积分数或/和升高柱温可实现两种免疫抑制剂的完全分离。

**图7:**
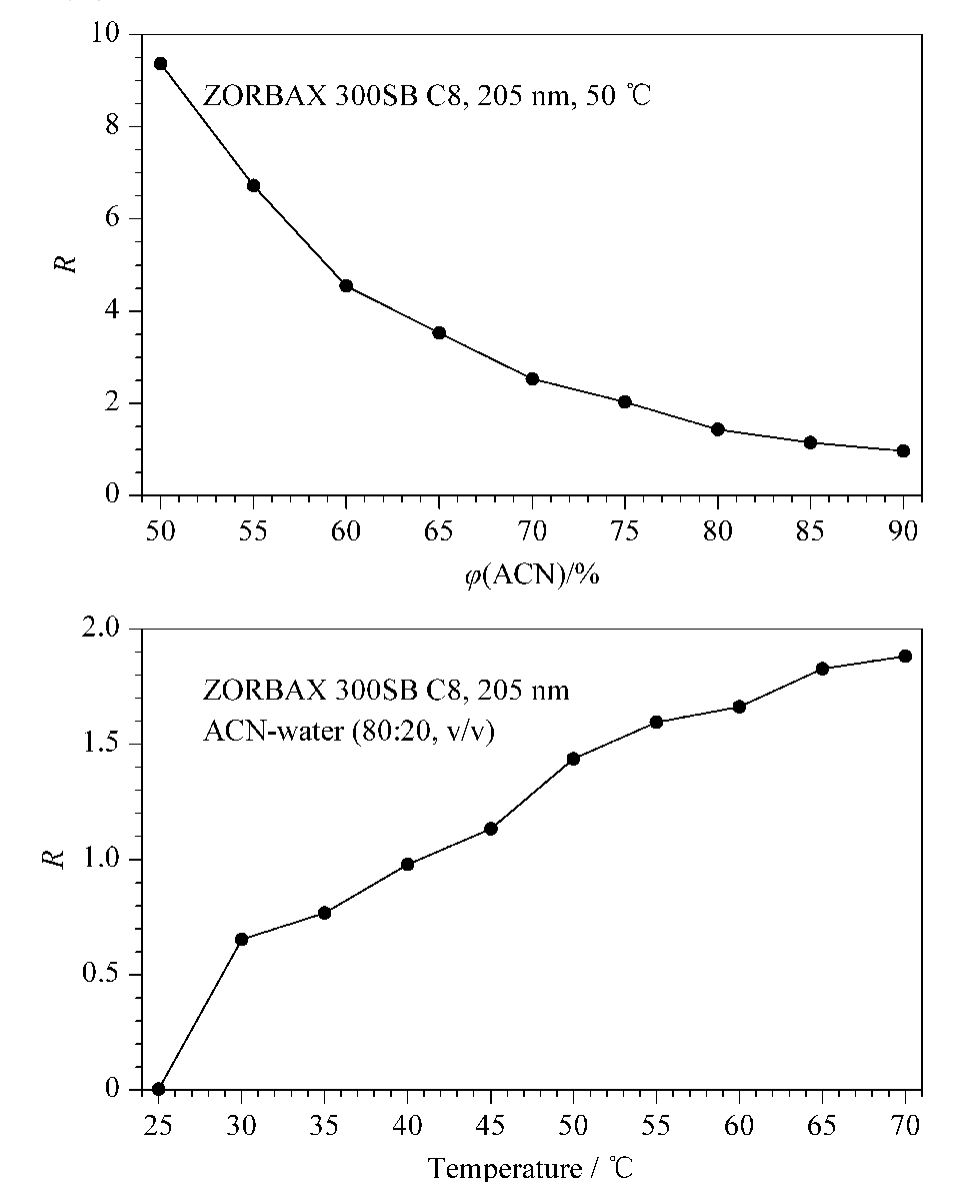
环孢素A和西罗莫司在不同色谱条件下的分离度

由于全血样品中含有大量的蛋白质和多肽，即使经过前处理仍可能会对这两种免疫抑制剂的检测产生干扰，因此，进一步考察了空白全血的基质效应，采用1.4节的液相色谱条件，对经1.3节前处理后的空白全血和混合标准储备溶液进行检测，色谱图见图8。结果表明，经前处理后的空白全血中，仍有内源性物质C1和C2可能会对这两种免疫抑制剂的检测产生干扰；当流动相为乙腈-水（70∶30， v/v）、柱温为60 ℃时，C1和C2对环孢素A的干扰较小，对西罗莫司的干扰几乎没有，且环孢素A与西罗莫司可在6 min内实现完全分离（*R*=3.7， 205 nm）。因此，兼顾两种免疫制剂的分离度、检测灵敏度和全血样品的基质效应，本文建立的快速分离和检测全血中两种免疫抑制剂的高效液相色谱分析条件具体见1.4节。

**图8:**
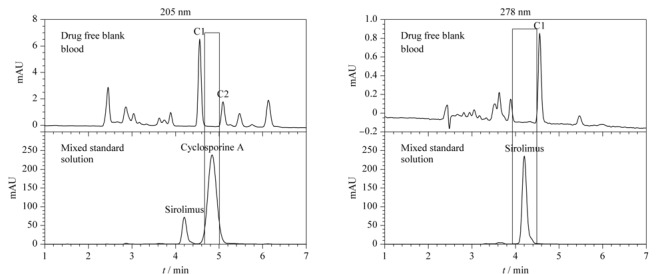
空白全血及环孢素A和西罗莫司混合标准储备溶液的色谱图

### 2.3 方法的线性范围、检出限和定量限

采用1.4节的液相色谱条件对1.2节配制的基质标准工作溶液进行测定，以分析物的峰面积（*Y*）和对应的质量浓度（*X*， ng/mL）进行线性回归，得到环孢素A和西罗莫司的线性方程和相关系数，以*S/N* ≥ 3和10的浓度为方法的检出限和定量限，结果见表2。结果表明，环孢素A和西罗莫司在一定范围内线性关系良好，相关系数均大于0.997，检出限分别为10 ng/mL和1 ng/mL，定量限分别为30 ng/mL和2 ng/mL。

**表2:** 环孢素A和西罗莫司的线性范围、线性方程、相关系数、检出限和定量限

Immunosuppressant drug	Linear range/（ng/mL）	Linear equation	*r*	LOD/（ng/mL）	LOQ/（ng/mL）
Cyclosporine A	30-2000	*Y*=0.053*X*+0.045	0.9976	10	30
Sirolimus	2-200	*Y*=0.098*X*+0.538	0.9989	1	2

*Y*：peak area；*X*：mass concentration，ng/mL.

### 2.4 精密度、准确度和加标回收率

在空白全血中，分别添加3个水平的环孢素A和西罗莫司溶液，进行精密度、准确度和加标回收率试验，加标样品按照1.3节方法处理后进行测定，结果见表3。结果显示，环孢素A和西罗莫司的平均回收率分别为83.5%~89.7%和95.8%~97.8%，相对标准偏差分别为3.2%~9.0%和3.4%~6.7%。该方法能够较好地满足全血样品中两种免疫抑制剂含量的测定要求。

**表3:** 空白全血样品中环孢素A和西罗莫司的加标回收率及相对标准偏差（*n*=5）

Immunosuppressant drug	Added/ （ng/mL）	Found/ （ng/mL）	Recovery/ %	RSD/ %
Cyclosporine A	30	25.06	83.5	9.0
	60	51.26	85.4	6.0
	300	269.01	89.7	3.2
Sirolimus	2	1.94	97.0	6.7
	4	3.83	95.8	4.2
	20	19.56	97.8	3.4

## 3 结论

本研究考察了环孢素A和西罗莫司在生物液相色谱柱和传统液相色谱柱上的色谱行为，并基于生物液相色谱柱建立了快速同时测定全血中这两种免疫抑制剂含量的高效液相色谱分析方法，该方法具有流动相简单、分析时间短、线性范围宽、灵敏度高等优点，可为全血中环孢素A和西罗莫司含量的检测提供技术支持。
